# Echogenicity as a surrogate for bioresorbable everolimus-eluting scaffold degradation: analysis at 1-, 3-, 6-, 12- 18, 24-, 30-, 36- and 42-month follow-up in a porcine model

**DOI:** 10.1007/s10554-015-0591-4

**Published:** 2015-01-28

**Authors:** Carlos M. Campos, Yuki Ishibashi, Jeroen Eggermont, Shimpei Nakatani, Yun Kyeong Cho, Jouke Dijkstra, Johan H. C. Reiber, Alexander Sheehy, Jennifer Lane, Marika Kamberi, Richard Rapoza, Laura Perkins, Hector M. Garcia-Garcia, Yoshinobu Onuma, Patrick W. Serruys

**Affiliations:** 1 Department of Interventional Cardiology, Thoraxcenter, Erasmus University Medical Centre, s-Gravendijkwal 230, 3015 CE Rotterdam, The Netherlands; 2 Heart Institute (InCor), University of São Paulo Medical School, Sao Paulo, Brazil; 3 Leiden University Medical Center, Leiden, The Netherlands; 4 Cardialysis, Rotterdam, The Netherlands; 5 Abbott Vascular, Santa Clara, CA USA; 6 International Centre for Circulatory Health, NHLI, Imperial College London, London, UK

**Keywords:** Absorb, Bioresorbable vascular scaffold, Degradation, Echogenicity, IVUS, Porcine

## Abstract

The objective of the study is to validate intravascular quantitative echogenicity as a surrogate for molecular weight assessment of poly-l-lactide-acid (PLLA) bioresorbable scaffold (Absorb BVS, Abbott Vascular, Santa Clara, California). We analyzed at 9 time points (from 1- to 42-month follow-up) a population of 40 pigs that received 97 Absorb scaffolds. The treated regions were analyzed by echogenicity using adventitia as reference, and were categorized as more (hyperechogenic or upperechogenic) or less bright (hypoechogenic) than the reference. The volumes of echogenicity categories were correlated with the measurements of molecular weight (Mw) by gel permeation chromatography. Scaffold struts appeared as high echogenic structures. The quantification of grey level intensity in the scaffold-vessel compartment had strong correlation with the scaffold Mw: hyperechogenicity (correlation coefficient = 0.75; *P* < 0.01), upperechogenicity (correlation coefficient = 0.63; *P* < 0.01) and hyper + upperechogenicity (correlation coefficient = 0.78; *P* < 0.01). In the linear regression, the R^2^ for high echogenicity and Mw was 0.57 for the combination of hyper and upper echogenicity. IVUS high intensity grey level quantification is correlated to Absorb BVS residual molecular weight and can be used as a surrogate for the monitoring of the degradation of semi-crystalline polymers scaffolds.

**Electronic supplementary material:**

The online version of this article (doi:10.1007/s10554-015-0591-4) contains supplementary material, which is available to authorized users.

## Impact on daily practice

Changes in bioresorbable vascular scaffolds (BRS), design and compositions may affect their degradation and loss of biomechanical characteristics (with the risk of late recoil) and may be associated with a second wave of arterial wall inflammation. Therefore, studying the BRS degradation is crucial to fully understand this technology. The present work validates echogenicity as a surrogate for polylactide scaffold degradation.

## Introduction

Bioresorbable vascular scaffolds (BRS) are a novel approach to the interventional treatment of coronary artery disease (CAD), providing short-term vascular scaffolding combined with drug-delivery capability. They may offer potential advantages compared to metallic drug-eluting stents (e.g. adaptive remodeling, restoration of vasomotion and late luminal enlargement). The so called 4th revolution in coronary artery disease revascularization steered extensive scientific research in BRS developments [1–3].

It has been shown that the designs and materials of BRS platforms—either metallic or polymeric—influence the resorption process [3–5]. Considering the variety of possible platforms, it is necessary to establish tools capable of monitoring the degradation process and its correlated mechanical characteristics.

Intravascular ultrasound-derived parameters have shown to be useful to assess the BRS resorption of metallic and polymeric scaffolds in humans [6–8]. One of the most studied intravascular ultrasound (IVUS) techniques to evaluate the resorption process is called differential echogenicity [8, 9]. This method consists in an automated and quantitative three-dimensional analysis of coronary tissue components scored for echogenicity using as reference the mean level of the adventitia brightness [9] where scaffold struts appear as bright hyperechogenic structures. In clinical studies, a continuous decrease of echogenicity over time has been shown in regions treated with BRS, being putatively correlated to BRS degradation [7, 8]. However, in serial human assessments, changes in the adventitia and plaque-media compartment of the treated regions during the follow-up period could possibly affect these interpretations [10–14].

The objectives of the current study were: (1) to describe a novel method of echogenicity for tissue analysis; (2) to evaluate its reproducibility; and (3) to assess its aptitude to assess the BRS degradation process through a direct correlation with the molecular weight (Mw) in a preclinical model using a drug-eluting poly-l-lactide-acid (PLLA) bioresorbable scaffold (Absorb BVS, Abbott Vascular, Santa Clara, California).

## Methods

### Study devices

The device used in the present preclinical study is the same used in Cohort B of the ABSORB clinical trial [15, 16]. Absorb is a balloon-expandable BRS that consists of a polymer backbone of Poly (L-lactide) (PLLA) coated with a thin layer of a 1:1 mixture of Poly-D, L-lactide (PDLLA) polymer with the antiproliferative drug everolimus to form an amorphous drug-eluting coating matrix containing 100 μg of everolimus/cm^2^ of scaffold [17].

### Experimental model

For validation purposes, we analyzed non-atherosclerotic Yorkshire-Landrace swine which had been implanted with Absorb BVS via femoral access according to published procedures [18]. Absorb sizes were matched to the vessel size at a target balloon-to-artery ratio of 1.0–1.1 (10 % overstretch). Each animal received a single Absorb (3.0 × 18 mm for 1-, 3-, and 6-month and 3.0x12 mm for 12- to 42-month) in 2 or 3 main coronary arteries. Forty pigs (98 arteries) underwent IVUS acquisition and were then euthanized at 1-month (n = 12 scaffolds), 3-(n = 12), 6-(n = 14), 12-(n = 12), 18-(n = 12), 24-(n = 12), 30-(n = 8), 36-(n = 8) or 42-months (n = 8). Each scaffold had quantification of polymer degradation by gel permeation chromatography (GPC). Experimental studies received protocol approval from the institutional animal care and use committee and were conducted in accordance with American Heart Association guidelines for pre-clinical research and the Guide for the Care and Use of Laboratory Animals (National Institutes of Health 2010).

### Gel permeation chromatography (GPC)

A previously reported GPC method, with a slightly modified sample extraction/purification process, was employed to investigate the degradation of polymer over time by evaluating the number-average molecular weight (Mn) of polymer in the Absorb [19]. In the present method, the extraction and purification of the polymer was repeated up to five times until the polymer was fully extracted from the tissue (i.e., the polymer signal in the last extract below the quantitation limit of 0.3 mg/mL). The samples were analyzed prior at 1-, 3-, 6-, 12-, 18-, 24-, 30-, 36- and 42-months after implantation.

### IVUS acquisition and analysis

**Fig. 1:**
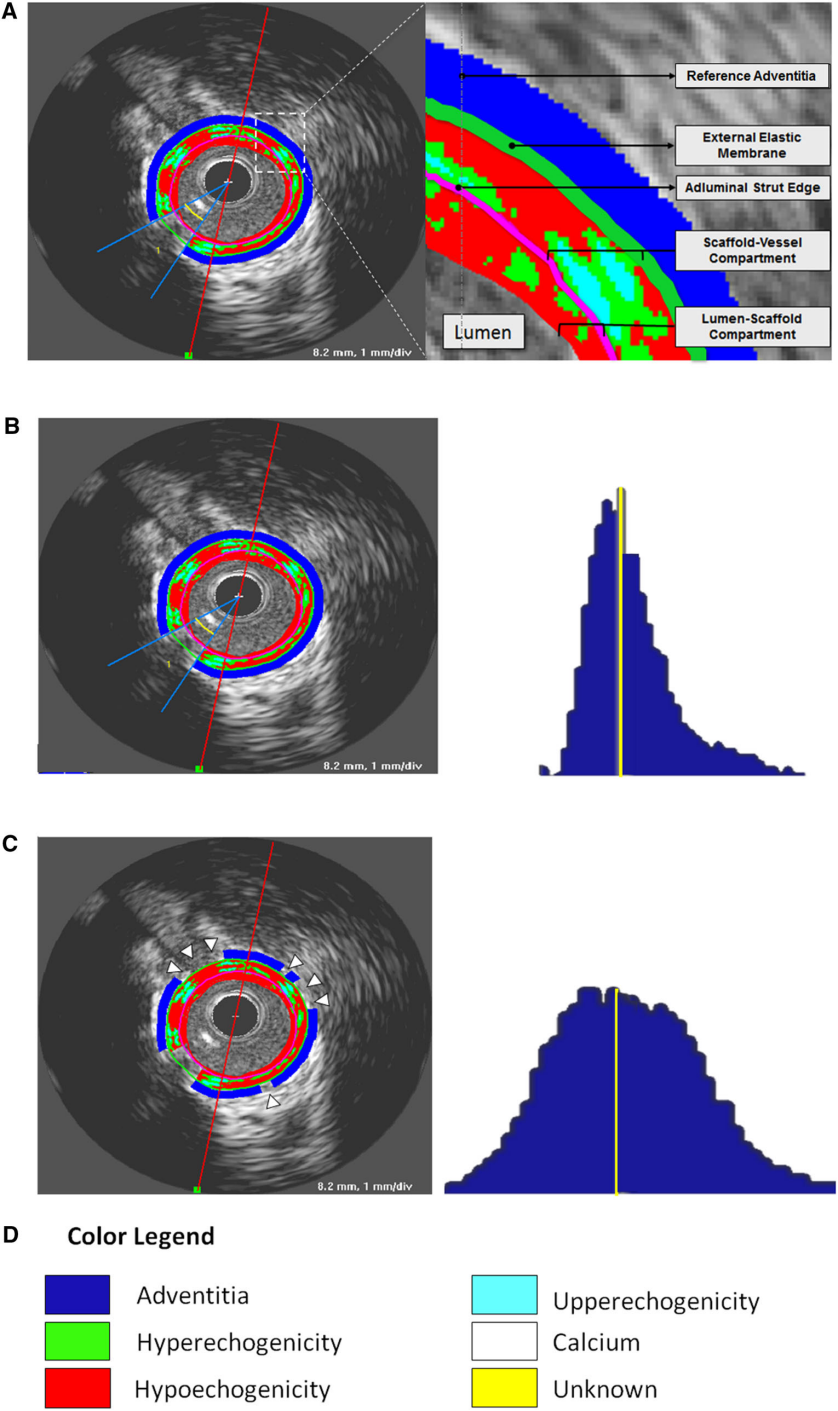
Differential echogenicity methodology. **a** The first step was to determine the lumen-scaffold and scaffold-vessel compartments by defining the vessel, lumen and luminal scaffold contours in every 0.5 mm. After guidewire masking, the software identifies the adventitia as a ring between 0.01 and 0.21 mm outside vessel contours. **b** However, if the software uses as reference the whole layer around the vessel contour, it will include low intensity structures (e.g., pericardium, side branches, low attenuated tissues, etc.) resulting in a histogram with a non-normal distribution (*right panel*). **c** The present software detects automatically high signal adventitia as reference, excluding low intensity structures (*arrow heads*). The *right panel* shows that the combination of high signal adventitia in all frames obtains a bell shaped normally distributed histogram. The yellow line represents the referential adventitial median value. **d** The color legend of each echogenicity classification is provided. As we used a non-atherosclerotic porcine model there was no calcification and unknown tissue. Nevertheless, the present software is able to detect theses tissues

All IVUS runs were acquired with 40 MHz mechanical systems, using Galaxy V2.02 (Boston Scientific, MA, USA) at 1-, 3-, 6- and 12-month follow-ups and iLab at 18-, 24-, 30-, 36- and 42-month (Boston Scientific, MA, USA). We used motorized pullback of 0.5 mm/s with a frame rate of 30 frames/second. The regions of interest were restrict to the scaffolded areas, identified by the first and the last cross-sectional IVUS frame in which scaffold struts could be identified and/or where the proximal or distal metallic markers could be identified. Vessel, scaffold and lumen contours were delimited every 0.5 mm blind to molecular weight results. We analysed four compartments by IVUS: the luminal, scaffold, vessel and the neointimal volume (vessel volume-lumen volume). The scaffold was delineated semiautomatically at the luminal leading edge of the struts and the lumen was delineated at the inner detectable tissue (Fig. 1).

To evaluate inter-observer reproducibility, 2 readers (C.C. and Y.I.) independently analyzed 30 segments randomly selected from the total number of the investigated segments. To determine intra-observer reproducibility, one reader (C.C.) analyzed these segments twice, with the second reading occurring 3 months later. The inter- and intra-observer reproducibility were good according to the conventional norms [20] (hyperechogenicity inter-observer interclass correlation coefficient [ICC] = 0.80, intra-observer ICC = 0.95; hypoechogenicity: inter-observer ICC = 0.78, intra-observer ICC = 0.97; upperechogenicity: inter-observer ICC = 0.92, intra-observer ICC = 0.97) (Supplementary material).

### Automatic quantitative echogenicity analysis

The principle of echogenicity has been previously described elsewhere [9, 21, 22]. Echogenicity aims to classify the vessel wall components located between the luminal boundary and the external elastic membrane (EEM) into categories based on their grey-level intensity in B-mode IVUS images rather than based on radiofrequency ultrasound signal analysis [23–26] (Fig. 1). Here we quantified 5 tissue types: hypoechogenic, hyperechogenic, calcified, upperechogenic and unknown.

Comparison with the adventitia allows for normalization with respect to transducer variability, gain settings and across populations [21]. However, in the analysis of atherosclerotic tissue, the adventitia can be partially obscured or darkened as a result of the guide-wire shadowing or the presence of dense tissue (e.g. calcium) which reduces the average grey-level values of the adventitia. Therefore, these parts need to be excluded from the reference adventitial area. To determine the reference adventitia area in each frame, the full adventitial area located just outside the EEM is first determined based on a minimum (0.01 mm) and maximum (0.21 mm) distance from the EEM contour (Fig. 1). To remove the low echogenic parts of the adventitia an adaptive threshold value for the entire adventitia area is determined based on Otsu’s method [27]. Otsu’s method is a classic automatic non-parametric threshold selection method which maximizes the between-class variance. Next, the adventitial area is divided into 2-degree wide sectors. If more than half of the pixels inside of a sector is below the adaptive threshold, the sector is excluded from the reference adventitia area. Finally, the histograms of the reference adventitial areas of the individual frames are combined into a global adventitia grey-level intensity histogram and the median value is computed as a threshold. Cross-section pixels with an intensity lower than the median value are classified as hypoechogenic, pixels with an intensity higher than the median value threshold are classified as hyperechogenic.

Calcified plaque is typically identified in B-mode IVUS images as a highly echogenic area creating an acoustic shadow [21]. To determine the high-intensity grey-level threshold for highly echogenic components we use the adaptive threshold selection method described in [28]. First Otsu’s method is applied to the entire grey-level histogram of an image resulting in an optimal threshold value. In the next 2 iterations, Otsu’s method is applied to the histogram of all intensities above the threshold found in the previous step. Next, we apply an in-house developed acoustic shadow detection algorithm. Highly echogenic areas with a grey-level intensity higher than the high-intensity threshold but without acoustic shadow behind them are classified as upperechogenic, while highly echogenic areas with acoustic shadow are classified as calcified and the shadow itself is classified as unknown. The entire method has been implemented and tested in QCU-CMS-Research v4.69 (research version of QIvus, developed by the Leiden University Medical Center) [29].

### Data analysis

Continuous variables are presented as mean ± SD or medians (interquartile range). The ANOVA test was used to compare continuous variables. As we had different scaffold lengths we normalized all measurements by the mean length for all pigs as described previously [30]. This adjusts for differing segment lengths across animals, thereby providing equal weighting of each individual in the calculation of echogenicity volumes. The residual scaffold molecular weight by GPC was compared to the echogenicity findings and the correlation coefficient was used as a measure of the degree of relationship (Pearson’s correlation coefficient). A linear regression was used to evaluate if hyper and/or upperechogenicity were able to predict the residual molecular weight. A hierarchical cluster analysis using Ward’s method (Squared Euclidean distance) was applied for hyper + upperechogenicity and hypoechogenicity volumes. The differences were regarded significant when *P* < 0.05 (two-tailed). SPSS version 21.0 (SPSS Inc., Chicago, Illinois) was used for all statistical analyses.

## Results

**Fig. 2:**
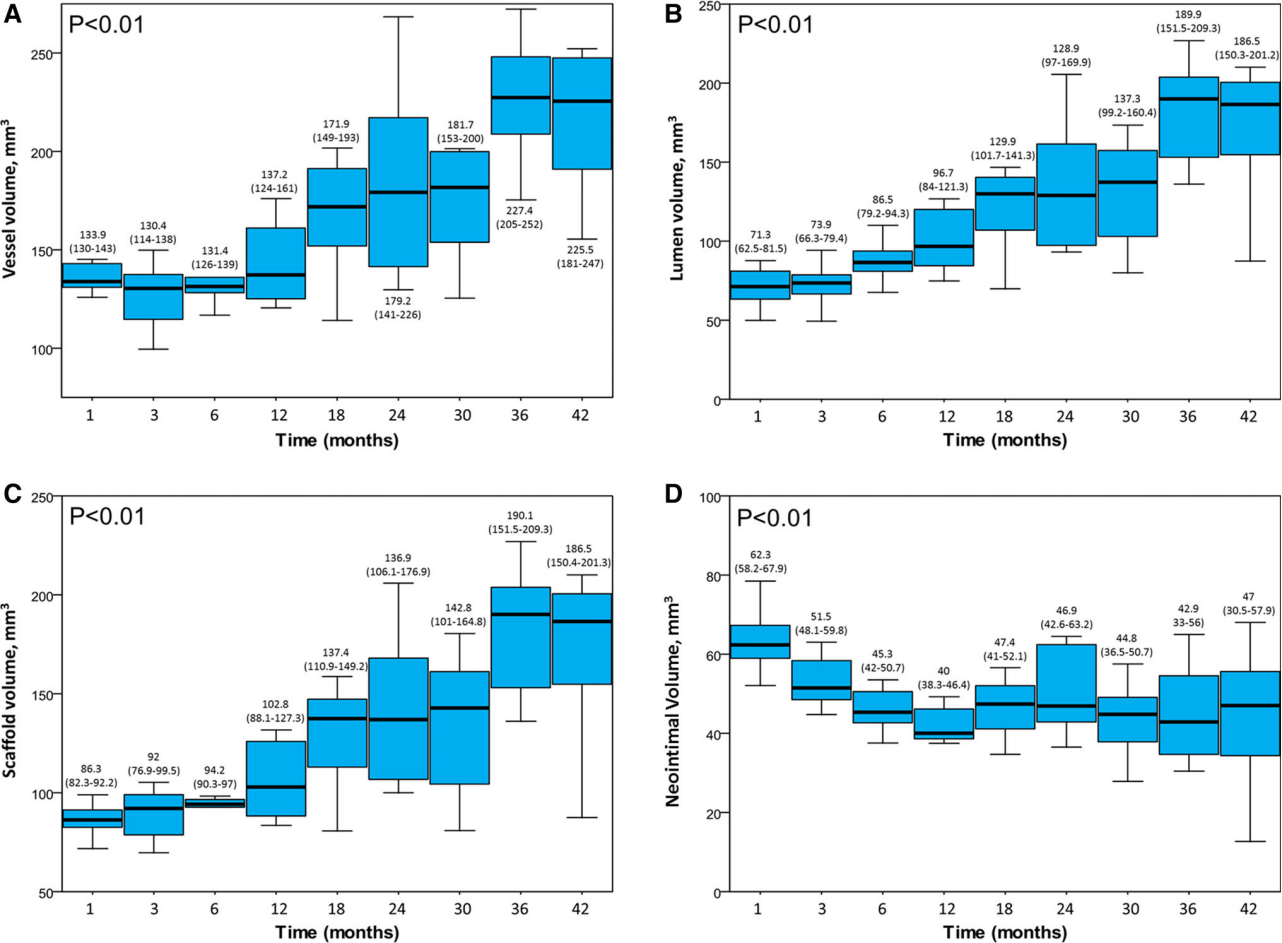
Grey Scale intravascular ultrasound volumetric findings at different time points. **a** Vessel volume; **b** Lumen Volume; **c** Scaffold Volume and **d** Neointimal Volume. Values are median and interquartile range

**Fig. 3:**
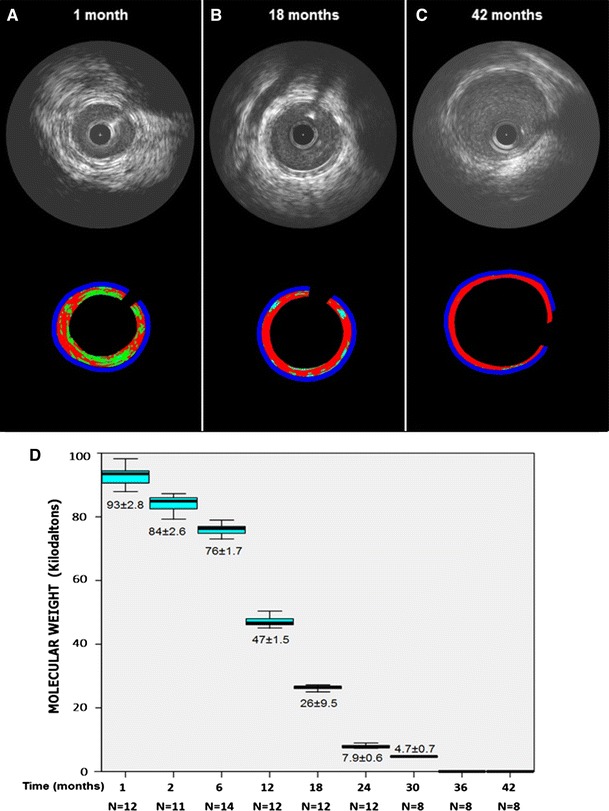
IVUS echogenicity analysis at 1- (**a**), 18- (**b**) and 42-month (**c**). The high echogenic (including hyper = *light green* and upper = *light blue*) parameters decrease over time. **d** Gel permeation chromatography (GPC) for the assessment of degradation of Absorb showing the in vivo degradation of polymer of Absorb over time

The main grey scale IVUS volumetric findings are shown in Fig. 2 and the comparisons between each group are given in the supplementary material (Tables 2-5). The mean scaffold length was 16.5 mm. Compared with 1-month follow-up, the vessel, scaffold and lumen volumes had a trend to be larger after 18-month follow-up. These three aforementioned volumes were significantly larger at 36- and 42-month. Additionally, the neointima had the biggest volume at 1-month follow-up, being similar among groups thereafter (Fig. 3).

### Differential echogenicity and molecular weight

Table 1 summarizes the main findings on differential echogenicity and mean Mw at each time point. The highest total hypoechogenicity volume was found at 1-month follow-up, the time point with also the highest neointimal hyperplasia as aforementioned. The lumen-scaffold compartment had an increase in hyper + upperechogenic volumes up to 12-month and subsequently a decrease until 42-month. Using the as reference the 1-month group, the hyper + upperechogenic decreased significantly in the scaffold vessel compartment after 12 months (supplementary material).

**Table 1:** Differential echogenicity findings and polymer molecular weight by gel permeation chromatography

	1 Month (n = 12)	3 Months (n = 12)	6 Months (n = 14)	12 Months (n = 12)	18 Months (n = 12)	24 Months (n = 12)	30 Months (n = 8)	36 Months (n = 8)	42 Months (n = 8)	*P* value for overall comparison
Total hypoechogenicity Volume (mm^3^)	49.3 ± 6.9	34.7 ± 6.8	31.4 ± 5.5	28.7 ± 4.5	33.3 ± 6.2	42.2 ± 8	28.4 ± 7	38.6 ± 12.2	37.6 ± 14.3	<0.01
% Total hypoechogenicity	79.4 ± 4.9	68.3 ± 7.8	70.3 ± 6.5	71.6 ± 6.7	80.5 ± 5.0	86.8 ± 5.4	78.1 ± 6.1	89.7 ± 4.3	92.0 ± 3.4	<0.01
Total hyperechogenicity volume (mm^3^)	8.5 ± 2.7	8.7 ± 4	6.6 ± 1	6.5 ± 2.8	4.8 ± 1	3.9 ± 1.5	4.3 ± 1.3	2.8 ± 1.2	2.2 ± 1.1	<0.01
% Total Hyperechogenicity	13.6 ± 3.6	17.2 ± 7.0	15.5 ± 2.7	16.1 ± 6.1	11.8 ± 2.4	8.0 ± 2.6	12.0 ± 2.9	6.7 ± 2.5	5.3 ± 1.9	<0.01
Total upperechogenicity volume (mm^3^)	6.3 ± 3.5	13.7 ± 5	12.9 ± 3.6	14.1 ± 7.1	9.5 ± 5.5	6.1 ± 3.6	11.3 ± 4.3	3.9 ± 2.1	2.9 ± 1.9	<0.01
% Total upperechogenicity	7.0 ± 3.9	14.5 ± 5.5	14.1 ± 5.3	12.3 ± 5.3	7.7 ± 4.0	5.2 ± 2.9	9.9 ± 3.4	3.7 ± 1.9	2.8 ± 1.6	<0.01
Total hyper and upperechogenicity volumes (mm^3^)	14.9 ± 4.5	22.4 ± 5.2	19.6 ± 4.4	20.6 ± 6.8	14.4 ± 6.1	10 ± 4.9	15.6 ± 5.3	6.7 ± 3.16	5.1 ± 3	<0.01
% Total hyper and upperechogenicity	20.6 ± 4.9	31.7 ± 7.8	29.7 ± 6.5	28.4 ± 6.7	19.5 ± 5.1	13.2 ± 5.4	21.9 ± 6.1	10.3 ± 4.3	8.1 ± 3.4	<0.01
Lumen-scaffold hypoechogenicity Volume (mm^3^)	13.6 ± 5.5	13.4 ± 4.3	7.7 ± 7.5	3.9 ± 1.9	6.7 ± 2.5	7.8 ± 4.1	2.9 ± 2	0 ± 0.1	0 ± 0.1	<0.01
% Lumen-scaffold hypoechogenicity	91.9 ± 4.3	88.8 ± 4.5	88.4 ± 4.1	80.5 ± 11.6	89.3 ± 3.7	92.3 ± 2.9	86.1 ± 4.5	22.0 ± 41.3	44.5 ± 48.3	<0.01
Lumen-scaffold hyperechogenicity volume (mm^3^)	1.25 ± 0.9	1.5 ± 0.8	1.2 ± 1.8	0.8 ± 0.5	0.4 ± 0.2	0.3 ± 0.2	0.3 ± 0.2	0	0	<0.01
% Lumen-scaffold hyperechogenicity	8.1 ± 4.4	10.7 ± 4.7	11.0 ± 3.8	19.0 ± 11.6	6.5 ± 3.0	4.0 ± 2.0	9.9 ± 3.5	1.8 ± 5.0	3.7 ± 8.7	<0.01
Lumen-scaffold upperechogenicity volume (mm^3^)	1.9 ± 1.3	6.6 ± 2.8	6.6 ± 2.8	9.3 ± 5.1	6.6 ± 3.9	3.8 ± 2.2	7.7 ± 3.22	2.4 ± 1.4	1.8 ± 1.3	<0.01
% Lumen-scaffold upperechogenicity	0.1 ± 0.1	0.5 ± 0.7	0.5 ± 0.7	0.4 ± 0.5	4.2 ± 0.9	3.1 ± 1.2	4.0 ± 1.7	1.2 ± 3.4	1.8 ± 51	<0.01
Lumen-scaffold hyper and upperechogenicity Volumes (mm^3^)	3.2 ± 1.5	8.1 ± 3.1	7.9 ± 2.6	10.1 ± 5.4	7.1 ± 4	4.1 ± 2.4	8 ± 3.3	2.4 ± 1.4	1.8 ± 1.3	<0.01
% Lumen-scaffold hyper and upperechogenicity	8.1 ± 4.3	11.2 ± 4.5	11.6 ± 4.1	19.5 ± 11.6	10.7 ± 37	7.1 ± 2.9	13.9 ± 4.5	3.0 ± 8.4	5.5 ± 10.3	<0.01
Scaffold-vessel hypoechogenicity volume (mm^3^)	35.7 ± 4	21.3 ± 5.1	23.8 ± 9.3	24.9 ± 4.6	26.6 ± 4.9	34.4 ± 7.9	25.5 ± 7.9	38.5 ± 12.2	37.7 ± 14.3	<0.01
% Scaffold-vessel hypoechogenicity	75.5 ± 6.0	59.6 ± 8.6	66.9 ± 8.3	70.1 ± 6.9	78.4 ± 6.2	85.3 ± 6.3	77.0 ± 6.3	89.7 ± 4.3	91.9 ± 3.4	<0.01
Scaffold-vessel hyperechogenicity volume (mm^3^)	7.3 ± 2	7.2 ± 4.2	5.4 ± 2.3	5.7 ± 2.5	4.4 ± 0.9	3.5 ± 1.3	4 ± 1.4	2.8 ± 1.1	2.2 ± 1.2	<0.01
% Scaffold-vessel hyperechogenicity	15.4 ± 4.1	19.5 ± 7.3	16.3 ± 2.9	15.9 ± 5.7	13.1 ± 2.8	8.9 ± 3.0	12.3 ± 3.0	6.7 ± 2.5	5.3 ± 1.9	<0.01
Scaffold-vessel upperechogenicity volume (mm^3^)	4.4 ± 2.6	7.1 ± 2.5	6.3 ± 1.8	4.8 ± 2.1	2.9 ± 1.7	2.3 ± 1.4	3.5 ± 1.5	1.5 ± 0.8	1.1 ± 0.8	<0.01
% Scaffold-vessel upperechogenicity	9.2 ± 5.3	20.8 ± 8.3	16.7 ± 6.7	14.4 ± 6.4	8.5 ± 4.9	5.8 ± 3.5	10.7 ± 3.7	3.7 ± 1.9	2.8 ± 1.6	<0.01
Scaffold-vessel hyper and upperechogenicity volumes (mm^3^)	11.7 ± 3.4	14.3 ± 3.8	11.7 ± 3.8	10.5 ± 2.4	7.3 ± 2.3	5.8 ± 2.7	7.5 ± 2.7	4.3 ± 1.9	3.2 ± 1.9	<0.01
% Scaffold-vessel hyper and Upperechogenicity	24.6 ± 6.0	40.4 ± 8.6	33.1 ± 8.3	29.9 ± 6.9	21.7 ± 6.2	14.7 ± 6.3	23.0 ± 6.3	10.4 ± 4.3	8.1 ± 3.4	<0.01
Molecular weight (kDa)	92.9 ± 2.8	84.2 ± 2.5	76 ± 1.7	47.1 ± 1.5	26.2 ± 0.9	7.9 ± 0.6	4.7 ± 73.9	0	0	<0.01

The GPC results indicated a continuous decrease in molecular weight over time. The rate of reduction was slower during the first 6-months of scaffold implantation followed by a more rapid decline thereafter, being fully resorbed 36-months after implantation (Fig. 2).

**Fig. 4:**
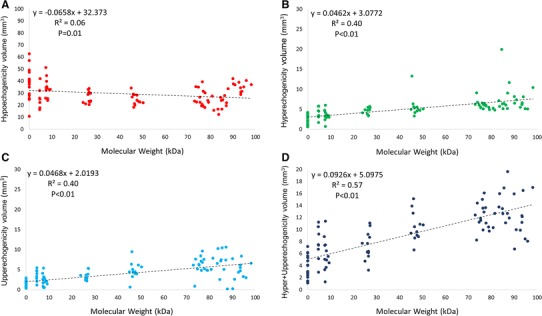
Linear regressions between molecular weight and echogenicity derived parameters in the scaffold-vessel compartment

To validate the scaffold degradation by echogenicity we took into consideration the hyper- and upperechogenicity in the scaffold-vessel compartment (Fig. 1). As shown in Table 1, the earlier IVUS were more likely to present higher grey-level intensity (hyper + upperechogenicity). The scaffold-vessel hyperechogenicity (Pearson correlation coefficient = 0.75; *P* < 0.01), upperechogenicity (Pearson correlation coefficient = 0.63; *P* < 0.01) and hyper + upperechogenicity (Pearson correlation coefficient = 0.78; *P* < 0.01) had strong correlation with the scaffold molecular weight. As shown in Fig. 4, in linear regression, the best correlation found in linear regression model for molecular weight was scaffold-vessel hyper + upperechogenicity (R squared = 0.57; *P* < 0.01); i.e., all grey-level intensity higher than median adventitia in the scaffold-vessel compartment should be considered for monitoring the degradation process of this semi-crystalline polymers scaffold. Post-Hoc comparisons between each group are given in the supplementary material (Tables 6-8).

**Fig. 5:**
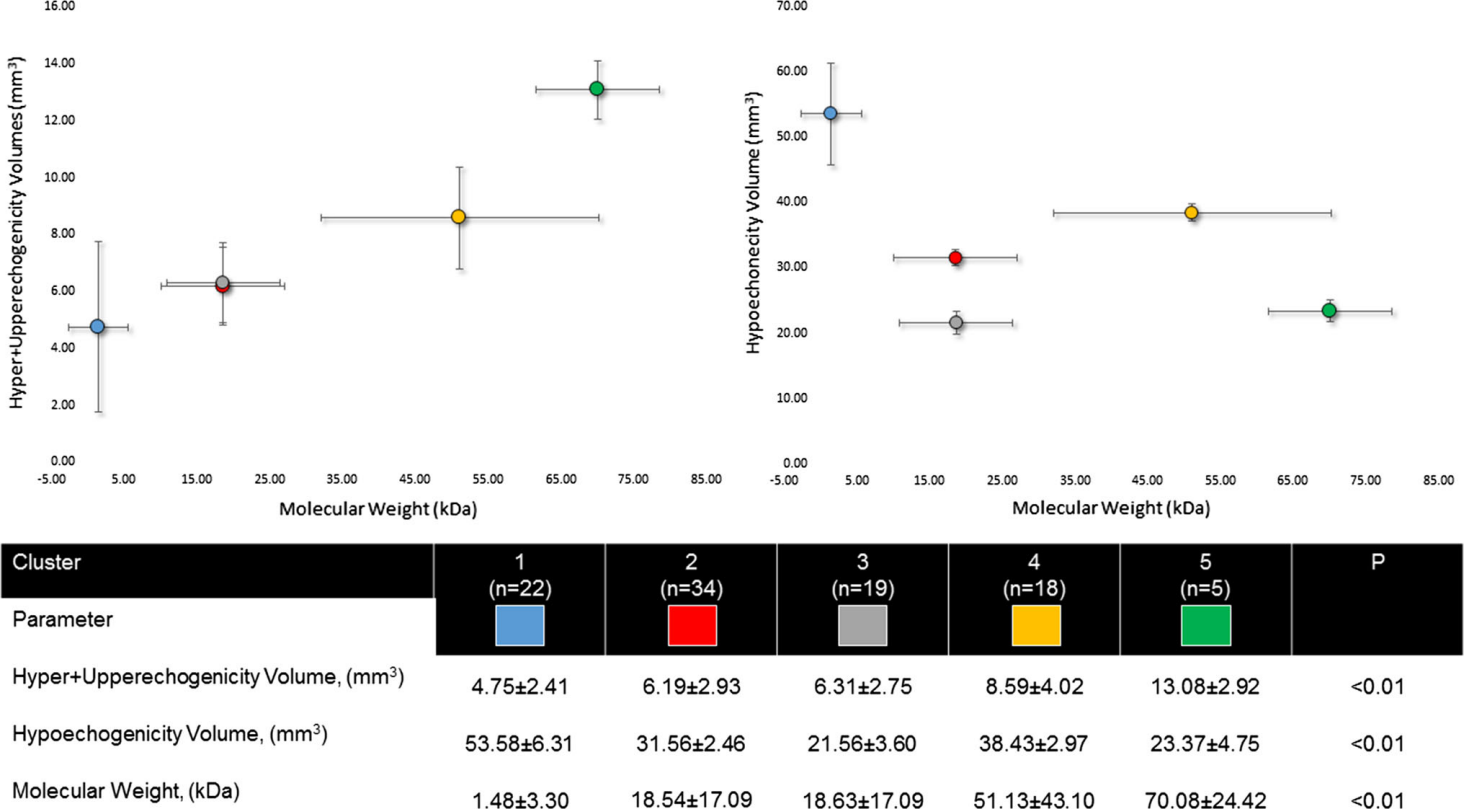
A hierarchical cluster analysis labeled by animal was run for scaffold-vessel hyper + upperechogenicity and hypoechogenicity. Cluster 2 and 3 had similar hyper + upperechogenicity but statistically significant greater hypoechogenicity volumes in the cluster 2. Cluster 3 and 5 had similar hypoechogenicity but markedly higher hyper + upperechogenicity volumes in cluster 5. There was a clear positive association between scaffold-vessel hyper + upperechogenicity and molecular weight. The sample sizes are number of scaffolds included in each pig cluster. The values are mean ± standard deviation and the errors bars are 95 % confidence interval

Additionally, a cluster analysis was run for scaffold-vessel hyper + upperechogenicity and hypoechogenicity. It produced five clusters, among which the variables were significantly different in the main (Fig. 5). The comparison among clusters of hyper + upperechogenicity showed a clear positive association scaffold-vessel hyper + upperechogenicity and molecular weight (Fig. 5).

## Discussion

In the present study, using IVUS grey scale derived parameters we attempted to assess the degradation process of the Absorb poly-L-lactide bioresorbable everolimus-eluting scaffold at multiple time points in a porcine model. The major findings of this study can be summarized as follows: (1) hyperechogenic and upperechogenic thresholds had strong and positive correlations with the scaffold molecular weight assessment; (2) the combination of hyper and upperechogenicity could be used as a surrogate for the chromatographic assessment of scaffold molecular weight and (3) echogenicity demonstrated good inter- and intra-observer reproducibility (Supplementary Material).

The present manuscript describes a new software designed to assess the differential echogenicity and, for the first time, ascertained the correlation between IVUS grey scale intensities and quantitative assessment of Mw by GPC. The first novelty is that it was not necessary to use ECG gating and therefore, it is not needed a dedicated IVUS console or post-processing correction. The robustness of this method and the aforementioned good reproducibility demonstrate, for the first time, good correlation of echogenicity with the degradation of the scaffold without being mandatory correction for motion artifacts [30].

Image resolution can be defined as the capability of making distinguishable the individual parts of an object. Therefore, the use of 40 MHz IVUS catheter in the present study has potential to be more precise to detect scaffold degradation than the previous methodology with the 20 MHz ultrasound [7, 31]. Ultrasound at a center frequency of 10 MHz has demonstrated to detect decline in the acoustic impedance of PLA when molecular weight varied from 60 to 24 kDa, but further decrease in molecular weight to 15 kDa did not result in discernible change [32]. In the present study, working with the higher resolution of the 40 MHz IVUS catheter, we were able to detect acoustic differences in 150 m thick samples degrading from ~100 to <4 kDa.

The use of ultrasound to monitor the degradation process of polymers has been initially proposed with a wave pulse-echo method in an in vitro essay [31]. Wu succeeded to monitor by ultrasound the degradation process of three biodegradable polymers: poly(glycolic acid) (PGA), poly(L-lactic acid) (PLLA) and 50:50 poly(D, L-lactide-co-glycolide) (PDLLG) [33]. Another IVUS based approach to detect the resorption process in human is virtual histology [6]. The spectral analysis of the raw backscattered ultrasound misrepresents polymeric struts as dense calcium (DC) and necrotic core (NC). As these parameters are shown to decrease over time after implantation, they have been correlated putatively with resorption [6, 16, 34, 35].

Previously, echogenicity has been used to assess paired serial acoustic properties of coronary plaques in BRS-treated segments in the clinical setting [7, 8, 35]. It has been shown that these segments had an increase in hyperechogenic tissue after implantation which decreased over time [7, 8, 35]. The aforementioned methodology succeeds to document the progressive decrease of high intensity grey level tissues in both metallic and polymeric BRS [8, 35].

However, until now, the link between echogenicity and the degradation process has been hypothetically assumed. The pending question was whether temporal plaque changes could interfere with the multistage degradation of the polymer and confound the echogenicity analysis. It has been shown that coronary atherosclerosis is a dynamic phenomenon and numerous factors can influence the atherosclerotic changes as detected by IVUS-derived parameters. For instance, statin treatment may reduce the percentage lipid volume index over time [13] and may increase the calcified plaque component [11]. Additionally, there is a significant decrease in NC (16 %) and DC (30 %) content in coronary plaque located behind the struts of the everolimus-eluting bioresorbable vascular scaffold [36]. All the above-mentioned confounding factors might influence the acoustic properties in the lumen-vessel compartment and hinder the clinical relevance of echogenicity for BRS degradation assessment.

As we have used a porcine non-atherosclerotic model, we did not have the confounding presence of coronary artery disease, thus enabling the evaluation of Poly-L-Lactide’s echogenic characteristics over time. Hyperechogenic, upperechogenenic and hyper + upperechogenic tissues had strong and positive correlations between echogenicity and the degradation process. Echogenicity is determined by the difference in acoustic impedance between two mediums, which is proportional to density and acoustic velocity. The acoustic velocity is proportional to the square root of the stiffness (bulk and shear moduli). Many factors impact the stiffness of PLA, including molecular weight, polydispersity, crystallinity, orientation of crystalline microstructure, and other environmental conditions [37]. As a result, one would expect to change the impedance of PLA as it degrades and molecular weight to have a generalized relationship to this decline.

Qualitatively, the correlation was however not perfectly linear. For instance, at 1-month the combination hyper + upper tended (without statistical significance; Table 6, supplementary material), in average, to be lower than at 3-months whereas the molecular weight had a continuous decrease in the same period. From the ultrasonic point of view, the significantly higher neointimal hyperplasia (Fig. 2) at 1-month might have affected the ultrasound penetration and therefore the echogenicity interpretation. Additionally, the scaffold-vessel hyper + upperechogenicity at 30-months was numerically comparable to that at 18-month. However, the degradation process may be influenced by individual biological factors and it has to be emphasized that these assessments were not serial. However, we showed a consistent individual positive correlation between the molecular weight and echogenicity (Figs. 4 and 5).

## Limitations

Arteries used for molecular weight assessment could not be evaluated histologically. Therefore, changes in the observed echogenicity (both lumen-scaffold and scaffold-vessel) could not be related to the histologic changes over time [19, 38]. As this study has been performed in a non-atherosclerotic model, it should be acknowledged that the rate of degradation has not been confirmed in atherosclerotic coronary arteries. However, as the degradation of PLLA is a hydrolytically driven and not enzymatically driven process, it is expected that the rates would be largely equivalent.

We could not test the reproducibility of the echogenicity IVUS findings in the two different consoles. However as we worked at the same ultrasound frequency (40 MHz) and the tissue classifications were normalized by the individual adventitia grey scale intensity we could show a robust correlation between scaffold degradation and high echogenic parameters. It has been shown that the comparison with the adventitia allows for normalization with respect to transducer variability, gain settings and across populations [21].

The changes in vessel, lumen, scaffold and neointima volumes over time are in line with the serial IVUS findings in the pre-clinical model and clinical setting showing progressive increase in vessel, lumen area and scaffold area [16, 18, 39, 40]. However, in the porcine model the somatic growth can influence our findings [39]. As we do not have the IVUS at baseline we could not normalize these geometrical changes for the increase in the reference vessel size. Nevertheless, this information has been described in the literature and are beyond the main scope of the current manuscript.

## Conclusion

IVUS high intensity grey level quantification is correlated to Absorb scaffold residual molecular weight assessment. Echogenicity is a reproducible technique which could be considered as a surrogate assessment of polylactide molecular weight decrease as assessed by chromatography and allows for monitoring of the degradation of semi-crystalline polymeric scaffolds.

## Electronic supplementary material

Below is the link to the electronic supplementary material.

Supplementary material 1 (DOCX 326 kb)

## Abbreviations

IVUS: Intravascular ultrasound
BRS: Bioresorbable scaffolds
PLLA: Poly-l-lactide-acid
PDLLA: Poly-D, L-lactide
Mw: Molecular weight
Absorb BVS: Poly-l-lactide-acid everolimus eluting bioresorbable scaffold
CAD: Coronary artery disease
