# Supplementary material for: Echogenicity as a surrogate for bioresorbable everolimus-eluting scaffold degradation: analysis at 1-, 3-, 6-, 12- 18, 24-, 30-, 36- and 42-month follow-up in a porcine model
Source: Int J Cardiovasc Imaging. 2015 Jan 28;31(3):471–82. doi: 10.1007/s10554-015-0591-4 (PMC4368838; doi:10.1007/s10554-015-0591-4)
Supplement: Supplementary file 1 — Supplementary material 1 (DOCX 326 kb) [file 10554_2015_591_MOESM1_ESM.docx]

**Supplementary Material**

***Statistical analysis***

The inter- and intra-observer reproducibility estimated with the interclass correlation coefficient (ICC). An ICC <0.4 indicates bad agreement, an ICC between 0.4 and 0.75 indicates moderate agreement, and ICC values >0.75 indicates good agreement. For the Bland–Altman analysis, both the absolute and relative difference (absolute difference divided by the mean value of both measurements) of the measurements were analyzed. The correlation between the different observers was analyzed by simple linear regression. The Bland-Altman plots were drawn for the scaffold-vessel hyper-, hypo- and upperechogenicity and the limit of agreement was defined as the mean ± 1.96 standard deviations of the absolute difference.

**Table 1.** **Inter- and Intra-observer Variability of hyper, hypo and upperechogenicity**

SD: standard deviation, CI: Confidence interval Dif.: difference, ICC: interclass correlation coefficient

|  |  |  |  | Intra-observer | | |  | Inter-observer | | |
| --- | --- | --- | --- | --- | --- | --- | --- | --- | --- | --- |
|  | Observer A  1^st^  Mean (SD) | Observer A  2^nd^  Mean (SD) | Observer B  Mean (SD) | Absolute Diff  (95% CI) | Relative Diff (%)  (95% CI) | ICC  (95% CI) |  | Absolute Diff  (95% CI) | Relative Diff (%)  (95% CI) | ICC  (95% CI) |
| Hypoechogenicity volume, mm^3^ | 29.9  (12.2) | 29.4  (10.7) | 25.9  (8.4) | 0.6  (-0.4 to 1.5) | 0  (-3 to 3) | 0.97  (0.95 to 0.98) |  | 4  (1.8 to 6.2) | 9  (4 to 15) | 0.78  (0.64-0.87) |
| Hiperechogenicity volume, mm^3^ | 4.9  (2.7) | 5.1  (2.2) | 4.9  (1.6) | -0.1  (-0.4 to 0.15) | -8  (-3 to -14) | 0.95  (0.92 to 0.97) |  | 0.1  (-0.4 to 0.6) | -9  (0 to -19) | 0.80  (0.72-0.87) |
| Upperechogenicity volume, mm^3^ | 4.2  (3.1) | 4.6  (2.9) | 4.5  (2.5) | 0.4  (-0.6 to -0.2) | - 10  (-1 to - 19) | 0.97  (0.95 to 0.98) |  | 0.3  (-0.1 to 0.7) | -6  (-13 to 8) | 0.92  (0.87-0.97) |

**Figure 1.** **The intra-observer reproducibility analysis**

The simple regression linear and Bland-Altman plot are shown: inter-observer reproducibility to assess the Scaffold-Vessel Hyperechogenicty (A) Scaffold-Vessel Hypoechogenicty and Scaffold-Vessel Upperechogenicty (C).


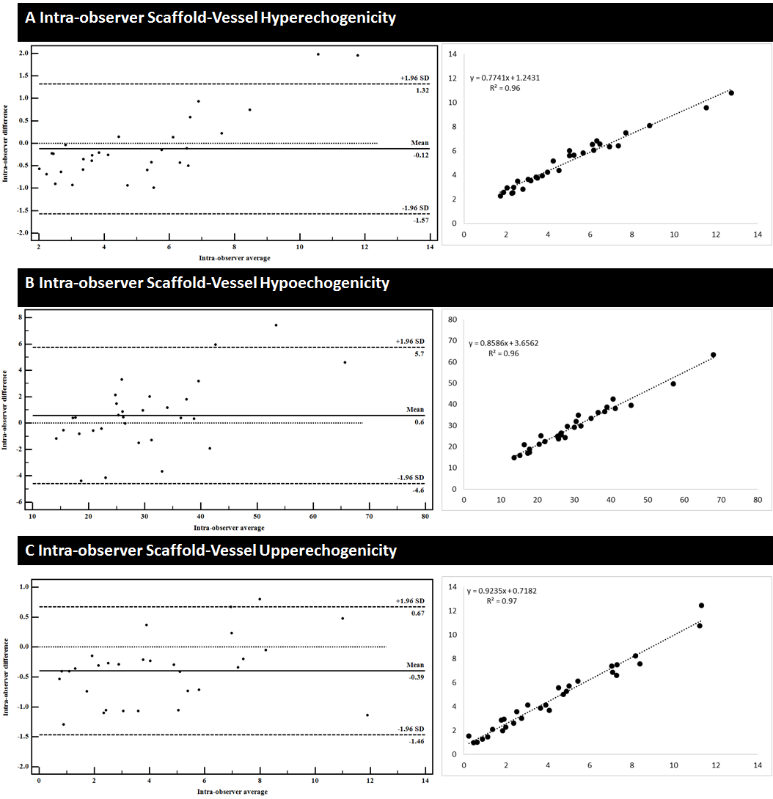


**Figure 2.** **The inter-observer reproducibility analysis**

The simple regression linear and Bland-Altman plot are shown: inter-observer reproducibility to assess the Scaffold-Vessel Hyperechogenicty (A) Scaffold-Vessel Hypoechogenicty and Scaffold-Vessel Upperechogenicty (C).


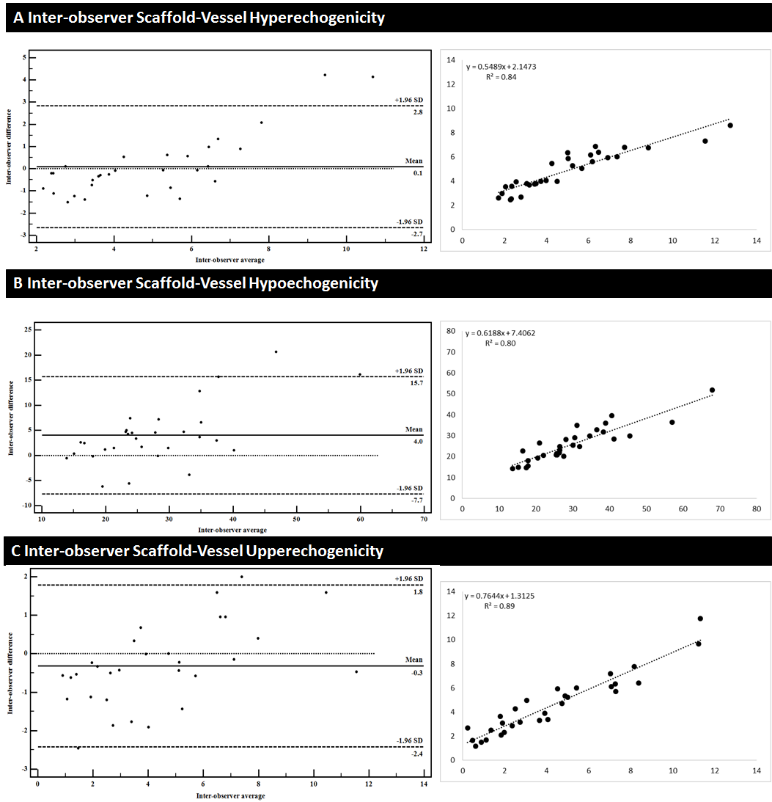


**Comparison of normalized grey scale intravascular ultrasound volumetric findings for each different time point**

The ANOVA with Tukey Post-Hoc test was used to compare continuous variables between groups.

**Table 2. Comparison of Vessel Volumes. Numbers are P values Between Groups**

|  | 1-month | 3-month | 6-month | 12-month | 18-month | 24-month | 30-month | 36-month | 42-month |
| --- | --- | --- | --- | --- | --- | --- | --- | --- | --- |
| 1-month |  | 0.99 | 1.00 | 1.00 | 0.09 | <0.01 | 0.05 | <0.01 | <0.01 |
| 3-month | 0.99 |  | 1.00 | 0.81 | 0.01 | <0.01 | <0.01 | <0.01 | <0.01 |
| 6-month | 1.00 | 1.00 |  | 0.99 | 0.04 | <0.01 | 0.02 | <0.01 | <0.01 |
| 12-month | 1.00 | 0.81 | 0.99 |  | 0.36 | 0.01 | 0.20 | <0.01 | <0.01 |
| 18-month | 0.09 | 0.01 | 0.04 | 0.36 |  | 0.81 | 1.00 | <0.01 | <0.01 |
| 24-month | <0.01 | <0.01 | <0.01 | 0.01 | 0.81 |  | 0.99 | 0.02 | 0.20 |
| 30-month | 0.05 | <0.01 | 0.02 | 0.20 | 1.00 | 0.99 |  | 0.01 | 0.05 |
| 36-month | <0.01 | <0.01 | <0.01 | <0.01 | <0.01 | 0.02 | 0.01 |  | 1.00 |
| 42-month | <0.01 | <0.01 | <0.01 | <0.01 | <0.01 | 0.20 | 0.05 | 1.00 |  |

**Table 3. Comparison of Lumen Volumes. Numbers are P values Between Groups**

|  | 1-month | 3-month | 6-month | 12-month | 18-month | 24-month | 30-month | 36-month | 42-month |
| --- | --- | --- | --- | --- | --- | --- | --- | --- | --- |
| 1-month |  | 1.00 | 0.79 | 0.13 | <0.01 | <0.01 | <0.01 | <0.01 | <0.01 |
| 3-month | 1.00 |  | 0.87 | 0.19 | <0.01 | <0.01 | <0.01 | <0.01 | <0.01 |
| 6-month | 0.79 | 0.87 |  | 0.94 | 0.04 | <0.01 | 0.01 | <0.01 | <0.01 |
| 12-month | 0.13 | 0.19 | 0.94 |  |  | 0.07 | 0.26 | <0.01 | <0.01 |
| 18-month | <0.01 | <0.01 | 0.04 | 0.61 | 0.61 |  | 1.00 | <0.01 | <0.01 |
| 24-month | <0.01 | <0.01 | <0.01 | 0.07 | 0.96 | 0.96 | 1.00 | <0.01 | 0.05 |
| 30-month | <0.01 | <0.01 | 0.01 | 0.26 | 1.00 | 1.00 |  | 0.01 | 0.06 |
| 36-month | <0.01 | <0.01 | <0.01 | <0.01 | <0.01 | <0.01 | 0.01 |  | 1.00 |
| 42-month | <0.01 | <0.01 | <0.01 | <0.01 | <0.01 | 0.05 | 0.06 | 1.00 |  |

**Table 4. Comparison of Scaffold Volumes. Numbers are P values Between Groups**

|  | 1-month | 3-month | 6-month | 12-month | 18-month | 24-month | 30-month | 36-month | 42-month |
| --- | --- | --- | --- | --- | --- | --- | --- | --- | --- |
| 1-month |  | 1.00 | 1.00 | 0.85 | 0.01 | <0.01 | 0.01 | <0.01 | <0.01 |
| 3-month | 1.00 |  | 1.00 | 0.77 | 0.01 | <0.01 | 0.01 | <0.01 | <0.01 |
| 6-month | 1.00 | 1.00 |  | 0.97 | 0.03 | <0.01 | 0.02 | <0.01 | <0.01 |
| 12-month | 0.85 | 0.77 | 0.97 |  | 0.42 | 0.03 | 0.30 | <0.01 | <0.01 |
| 18-month | 0.01 | 0.01 | 0.03 | 0.42 |  | 0.95 | 1.00 | <0.01 | 0.01 |
| 24-month | <0.01 | <0.01 | <0.01 | 0.03 | 0.95 |  | 1.00 | 0.03 | 0.24 |
| 30-month | 0.01 | 0.01 | 0.02 | 0.30 | 1.00 | 1.00 |  | 0.01 | 0.10 |
| 36-month | <0.01 | <0.01 | <0.01 | <0.01 | <0.01 | 0.03 | 0.01 |  | 1.00 |
| 42-month | <0.01 | <0.01 | <0.01 | <0.01 | 0.01 | 0.24 | 0.10 | 1.00 |  |

**Table 5. Comparison of Neointimal Volumes. Numbers are P values Between Groups**

|  | 1-month | 3-month | 6-month | 12-month | 18-month | 24-month | 30-month | 36-month | 42-month |
| --- | --- | --- | --- | --- | --- | --- | --- | --- | --- |
| 1-month |  | 0.07 | <0.01 | <0.01 | <0.01 | 0.01 | <0.01 | <0.01 | <0.01 |
| 3-month | 0.07 |  | 0.58 | 0.11 | 0.70 | 1.00 | 0.41 | 0.62 | 0.53 |
| 6-month | <0.01 | 0.58 |  | 0.98 | 1.00 | 0.90 | 1.00 | 1.00 | 1.00 |
| 12-month | <0.01 | 0.11 | 0.98 |  | 0.97 | 0.34 | 1.00 | 1.00 | 1.00 |
| 18-month | <0.01 | 0.70 | 1.00 | 0.97 |  | 0.95 | 1.00 | 1.00 | 1.00 |
| 24-month | 0.01 | 1.00 | 0.90 | 0.34 | 0.95 |  | 0.74 | 0.90 | 0.83 |
| 30-month | <0.01 | 0.41 | 1.00 | 1.00 | 1.00 | 0.74 |  | 1.00 | 1.00 |
| 36-month | <0.01 | 0.62 | 1.00 | 1.00 | 1.00 | 0.90 | 1.00 |  | 1.00 |
| 42-month | <0.01 | 0.53 | 1.00 | 1.00 | 1.00 | 0.83 | 1.00 | 1.00 |  |

**Comparison of scaffold-vessel echogenicity normalized intravascular ultrasound volumetric findings for each different time point**

The ANOVA with Tukey Post-Hoc test was used to compare continuous variables between groups.

**Table 6. Comparison of scaffold-vessel normalized Hiper+upperechogenicity Volumes. Numbers are P values Between Groups**

|  | 1-month | 3-month | 6-month | 12-month | 18-month | 24-month | 30-month | 36-month | 42-month |
| --- | --- | --- | --- | --- | --- | --- | --- | --- | --- |
| 1-month |  | 0.32 | 1.00 | 0.98 | <0.01 | <0.01 | 0.04 | <0.01 | <0.01 |
| 3-month | 0.32 |  | 0.65 | 0.03 | <0.01 | <0.01 | <0.01 | <0.01 | <0.01 |
| 6-month | 1.00 | 0.65 |  | 0.75 | <0.01 | <0.01 | <0.01 | <0.01 | <0.01 |
| 12-month | 0.98 | 0.03 | 0.75 |  | 0.10 | <0.01 | 0.32 | <0.01 | <0.01 |
| 18-month | <0.01 | <0.01 | <0.01 | 0.10 |  | 0.92 | 1.00 | 0.28 | 0.04 |
| 24-month | <0.01 | <0.01 | <0.01 | <0.01 | 0.92 |  | 0.89 | 0.95 | 0.49 |
| 30-month | 0.04 | <0.01 | <0.01 | 0.32 | 1.00 | 0.89 |  | 0.28 | 0.05 |
| 36-month | <0.01 | <0.01 | <0.01 | <0.01 | 0.28 | 0.95 | 0.28 |  | 1.00 |
| 42-month | <0.01 | <0.01 | <0.01 | <0.01 | 0.04 | 0.49 | 0.05 | 1.00 |  |

**Table 7. Comparison of scaffold-vessel normalized Hipoechogenicity Volumes. Numbers are P values Between Groups**

|  | 1-month | 3-month | 6-month | 12-month | 18-month | 24-month | 30-month | 36-month | 42-month |
| --- | --- | --- | --- | --- | --- | --- | --- | --- | --- |
| 1-month |  | 1.00 | 0.02 | <0.01 | <0.01 | 0.03 | <0.01 | <0.01 | <0.01 |
| 3-month | 1.00 |  | 0.03 | <0.01 | <0.01 | 0.04 | <0.01 | <0.01 | <0.01 |
| 6-month | 0.02 | 0.03 |  | <0.37 | 1.00 | 1.00 | 0.23 | <0.01 | <0.01 |
| 12-month | <0.01 | <0.01 | 0.37 |  | 0.78 | 0.38 | 1.00 | 0.56 | 0.56 |
| 18-month | <0.01 | <0.01 | 1.00 | 0.78 |  | 1.00 | 0.58 | 0.02 | 0.02 |
| 24-month | 0.03 | 0.04 | 1.00 | 0.38 | 1.00 |  | 0.24 | <0.01 | <0.01 |
| 30-month | <0.01 | <0.01 | 0.23 | 1.00 | 0.58 | 0.24 |  |  | 0.91 |
| 36-month | <0.01 | <0.01 | <0.01 | 0.56 | 0.03 | <0.01 | 0.91 | 0.91 | 1.00 |
| 42-month | <0.01 | <0.01 | <0.01 | 0.56 | 0.03 | <0.01 | 0.91 | 1.00 |  |

**Table 8. Comparison of scaffold-vessel normalized Hiperechogenicity Volumes. Numbers are P values Between Groups**

|  | 1-month | 3-month | 6-month | 12-month | 18-month | 24-month | 30-month | 36-month | 42-month |
| --- | --- | --- | --- | --- | --- | --- | --- | --- | --- |
| 1-month |  | 1.00 | 1.00 | 0.95 | 0.33 | 0.18 | 0.28 | 0.05 | 0.04 |
| 3-month | 1.00 |  | 0.99 | 0.52 | 0.05 | 0.02 | 0.05 | <0.01 | <0.01 |
| 6-month | 1.00 | 0.99 |  | 0.96 | 0.33 | 0.18 | 0.29 | 0.05 | 0.04 |
| 12-month | 0.95 | 0.52 | 0.96 |  | 0.97 | 0.89 | 0.92 | 0.46 | 0.46 |
| 18-month | 0.33 | 0.05 | 0.33 | 0.97 |  | 1.00 | 1.00 | 0.97 | 0.97 |
| 24-month | 0.18 | 0.02 | 0.18 | 0.89 | 1.00 |  | 1.00 | 1.00 | 1.00 |
| 30-month | 0.28 | 0.05 | 0.29 | 0.92 | 1.00 | 1.00 |  | 1.00 | 1.00 |
| 36-month | 0.05 | <0.01 | 0.05 | 0.47 | 0.97 | 1.00 | 1.00 |  | 1.00 |
| 42-month | 0.04 | <0.01 | 0.04 | 0.46 | 0.97 | 1.00 | 1.00 | 1.00 |  |

**Table 8. Comparison of scaffold-vessel normalized Upperechogenicity Volumes. Numbers are P values Between Groups**

|  | 1-month | 3-month | 6-month | 12-month | 18-month | 24-month | 30-month | 36-month | 42-month |
| --- | --- | --- | --- | --- | --- | --- | --- | --- | --- |
| 1-month |  | <0.01 | <0.01 | <0.01 | <0.01 | 0.83 | <0.01 | 1.00 | 1.00 |
| 3-month | <0.01 |  | 1.00 | 0.41 | 1.00 | 0.35 | 1.00 | 0.07 | 0.02 |
| 6-month | <0.01 | 1.00 |  | 0.38 | 1.00 | 0.28 | 1.00 | 0.05 | 0.01 |
| 12-month | <0.01 | 0.41 | 0.38 |  | 0.43 | <0.01 | 0.97 | <0.01 | <0.01 |
| 18-month | <0.01 | 1.00 | 1.00 | 0.43 |  | 0.37 | 1.00 | 0.06 | 0.02 |
| 24-month | 0.83 | 0.35 | 0.28 | <0.01 | 0.37 |  | 0.10 | 0.99 | 0.87 |
| 30-month | <0.01 | 1.00 | 1.00 | 0.97 | 1.00 | 0.10 |  | 0.02 | <0.01 |
| 36-month | 1.00 | 0.07 | 0.05 | <0.01 | 0.06 | 0.99 | 0.02 |  | 1.00 |
| 42-month | 1.00 | 0.02 | 0.01 | <0.01 | 0.02 | 0.87 | <0.01 | 1.00 |  |
